# Unveiling the role of air pollution in diabetic kidney disease: an integrated study combining network toxicology, machine learning, and Mendelian randomization

**DOI:** 10.1080/0886022X.2026.2668270

**Published:** 2026-05-24

**Authors:** Yi Kang, Qian Jin, Size Li, Yaping Lv, Xuezhe Wang, Xinyan Jin, Mengqi Zhou, Danwen Li, Jie Lv, Huijuan Zheng, Yaoxian Wang

**Affiliations:** a Dongzhimen Hospital, Beijing University of Chinese Medicine, Beijing, China; b Beijing University of Chinese Medicine School of Traditional Chinese Medicine, Beijing, China; c Renal Research Institution of Beijing University of Chinese Medicine, Beijing, China; d The Department of Laboratory Medicine, Renji Hospital, Affiliated to Shanghai Jiao Tong University School of Medicine, Shanghai, China; e Department of Traditional Chinese Medicine, Beijing Puren Hospital, Beijing, China

**Keywords:** Air pollution, network toxicology, diabetic kidney disease, machine learning, molecular docking, Mendelian randomization

## Abstract

Diabetic kidney disease (DKD) is a major and severe complication associated with diabetes. Air pollution is not only an independent risk factor for metabolic disorders but also an “accelerator” of DKD progression. This study seeks to investigate the molecular pathways connecting air pollution to DKD. Multiple databases were integrated to obtain potential target genes of 10 common air pollutants. The gene expression omnibus (GEO) database was employed to acquire DKD datasets. Differential expression analysis and weighted correlation network analysis (WGCNA) were performed to identify DKD-related genes. 12 machine learning algorithms were utilized to generate 113 unique predictive models, which were employed to select hub genes. Subsequently, MR, single-cell, gene set enrichment analysis (GSEA), and immune infiltration analyses were performed, followed by molecular docking of hub genes with air pollutants. A total of 714 targets were identified from 10 air pollutants, and 80 potential targets were identified from DKD transcriptomic data. Machine learning methods identified 5 hub genes that are closely associated with DKD. Mendelian randomization (MR) analysis indicated that, among the five hub genes, only NOS3 demonstrated a statistically significant causal association with DKD. Immune infiltration analysis found that hub genes were closely related to immune cells. Molecular docking validation indicated that certain air pollutants can stably bind with hub genes such as NOS3 and PTGS2. Air pollutants may be linked to alterations in various biological processes, potentially involving key genes such as ADH5, CASP3, NOS3, PTGS2, and SDHB, including potential metabolic reprogramming, inflammatory processes, and immune microenvironment changes.

## 1. Introduction

Diabetes is a persistent and long-term metabolic disorder. According to epidemiological data from the 2025 edition of the International Diabetes Federation, approximately 1 in 9 adults (aged 20–79) worldwide have diabetes, and this prevalence is projected to keep increasing in the years ahead [1]. It is noteworthy that approximately 40% of individuals with diabetes will develop diabetic kidney disease (DKD) [2]. In contrast to other complications associated with diabetes, the incidence of DKD has remained steady over the last three decades, and it has emerged as the primary cause of ESRD. Even with rigorous management of blood glucose and blood pressure, the progression of the disease may not be halted or slowed [3]. This situation suggests that there may be pathogenic mechanisms and potential risk factors for DKD that are not yet fully understood, and there is an urgent need for in-depth exploration through multidimensional research.

Air pollution is regarded as the most significant environmental risk factor contributing to non-communicable diseases. Approximately 99% of the global population is continuously exposed to polluted environments exceeding the limits set by the World Health Organization’s Global Air Quality Guidelines, and this persistent exposure poses a significant public health threat [4]. Previous studies primarily focused on the impact of air pollution on cardiovascular and pulmonary diseases [5,6]. Recent research has revealed that long-term exposure to high levels of air pollution is significantly associated with an increased risk of diabetes [7,8] and kidney diseases [9–11]. DKD is recognized as a significant complication linked to air pollution exposure in individuals with diabetes [12]. Air pollutants such as PM2.5 significantly increase UACR levels in diabetic patients [13] and exacerbate kidney function deterioration [14–16]. However, the toxic pathways and molecular mechanisms of air pollutants in the onset and progression of DKD remain unclear.

**Figure 1.**
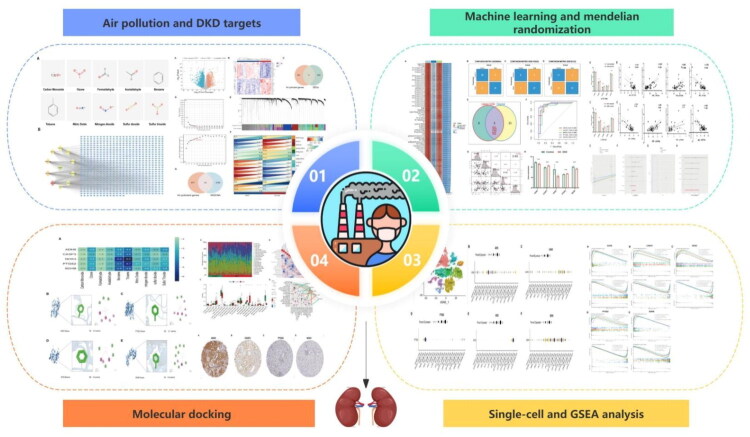
Research flowchart.

Traditional research methods struggle to comprehensively reveal the multi-target mechanisms in complex biological systems. Network toxicology offers a new research paradigm for studying the relationship between complex environmental exposures and diseases [17]. Machine learning algorithms can extract key features from complex biological big data, enhancing the accuracy and efficiency of identifying key molecules. This study integrates target information of 10 common air pollutants with DKD-related transcriptomic data to systematically screen and validate the key genes and potential molecular mechanisms by which air pollutants influence DKD. This research provides molecular-level evidence for understanding the relationship between air pollution and DKD. It also provides new insights and potential targets for the prevention and treatment of DKD, providing an innovative paradigm for precision diagnosis and treatment in environmental medicine. The detailed procedure of the study is illustrated in Figure 1.

## 2. Material and methods

### 2.1. Selection of air pollutants

This study selects ten major pollutants constituting environmental PM2.5 (CO, O_3_, CH_2_O, C_2_H_4_O, C_6_H_6_, C_7_H_8_, NO, NO_2_, SO_2_, SO_3_) based on pollutant emissions and health impacts. Among them, CO, O_3_, NO_2_, and SO_2_ are gaseous pollutants explicitly listed as priority control pollutants in the 2021 edition of the WHO Air Quality Guidelines. CO, O_3_, VOCs (such as CH_2_O, C_2_H_4_O, C_6_H_6_, C_7_H_8_), NOx, and SO_2_ are high-exposure, highly pathogenic air pollutants in both indoor and outdoor environments [18–25].

### 2.2. Air pollutant-related targets

Chemical structures of 10 air pollutants were obtained from the PubChem database. Human target genes for these pollutants were identified using TargetNet, Swiss Target Prediction (probability > 0), and the ChEMBL database. The target genes from these sources were combined and deduplicated to determine air pollution-related genes (ARGs).

### 2.3. DKD-related targets

The GSE96804 dataset (GPL17586) from the GEO database, comprising kidney tissue transcriptomic data from 41 DKD patients and 20 healthy individuals, was analyzed. Differential expression analysis was conducted using the limma package with thresholds of fold change (FC) > 1.2 and _adj_*P* < 0.05. Volcano plots and heatmaps were created using ggplot2 and pheatmap packages, respectively. Weighted gene co-expression network analysis (WGCNA) was used to construct co-expression networks and identify gene modules; genes from strongly correlated modules (*p* < 0.05) were selected.

### 2.4. PPI network construction and enrichment analysis

The differentially expressed genes (DEGs) and key module genes were cross-referenced with ARGs to identify potential targets through which air pollutants may impact DKD. These targets were uploaded to the STRING database to build a PPI network, and gene ontology (GO) and kyoto encyclopedia of genes and genomes (KEGG) enrichment analyses were conducted using the clusterProfiler package.

### 2.5. Machine learning screens hub genes

We employed twelve machine learning algorithms, including regularization methods (Lasso, Ridge, ElasticNet), generalized linear models (Stepglm, glmBoost, plsRglm), ensemble learning techniques (Random Forest, GBM, XGBoost), and pattern recognition models (SVM, LDA, Naïve Bayes). To mitigate overfitting, feature selection and model training were fully nested within a stratified 10-fold cross-validation framework. Within each training fold, feature selection was conducted independently, and the corresponding validation fold remained entirely unseen, thus preventing any potential information leakage. Furthermore, variance was controlled through the application of regularization methods (Lasso, Ridge, ElasticNet) and by constraining model complexity. Recursive feature elimination (RFE) was applied for variable ranking and selection. During the preliminary screening stage, Lasso, Random Forest, Stepglm, and glmBoost were used to narrow down the gene set, after which the remaining eight algorithms were used to construct predictive models. This process yielded 113 distinct model combinations, each subjected to hyperparameter optimization within the cross-validation framework. Model performance was principally evaluated using the area under the receiver operating characteristic curve (AUC). The model with the highest average AUC across the training cohort (GSE96804) and the independent test cohorts (GSE30122 and GSE142025) was ultimately selected as the optimal predictive model [26,27]. The hub genes were validated through the Nephroseq database (http://v5.nephroseq.org/), and their correlation with creatinine levels was assessed. The pROC package was used to calculate AUC to evaluate the predictive performance.

### 2.6. Mendel randomization(MR) analysis

A two-sample MR approach was employed to explore the causal link between NOS3 gene expression and DKD. Expression quantitative trait locus (eQTL) data for the NOS3 gene expression was obtained from the eQTLGen consortium public database (including blood samples from 31,684 individuals of European descent), and outcome data were sourced from the FinnGen R12 diabetic nephropathy dataset (finngen_R12_DM_NEPHROPATHY_EXMORE). SNPs associated with NOS3 expression were chosen as instrumental variables, and harmonization of data was carried out to guarantee consistency in allele coding. This process required the inclusion of no fewer than three independent SNPs, each demonstrating an F-statistic > 10. Multiple MR methods (IVW, MR-Egger, weighted median, and weighted mode) were used. Heterogeneity testing (Cochran’s Q) and pleiotropy assessment (MR-Egger intercept, MR-PRESSO) were performed, and sensitivity analysis was conducted using the leave-one-out method. MR analysis was performed using the TwoSampleMR and MRPRESSO packages in R software.

### 2.7. Single-cell analysis

Using single-cell sequencing data from both DKD patients and healthy individuals, obtained from the kidney integration transcriptomics (K.I.T.) database. Wilson et al. [28] conducted unbiased snRNA-seq on preserved human diabetic kidney samples. Based on this database, the present study explored the single-cell sequencing data of hub genes from diabetic nephropathy lesions and visualized the results.

### 2.8. Gene set enrichment analysis (GSEA) analysis

The “c2.cp.v7.2.symbols.gmt” reference gene set was obtained from the MSigDB database [29]. Following enrichment analysis, GSEA was visualized using the clusterProfiler package [30].

### 2.9. Immune infiltration analysis

The CIBERSORT algorithm was applied to assess immune cell infiltration levels between DKD and control samples, with the “PERM” parameter set to 100 and the significance threshold set at a *P* value of 0.05. Correlation heatmaps were generated using the corrplot package to visualize the relationships between hub genes and immune cells.

### 2.10. Verification of the expression pattern

To further confirm the expression patterns of hub genes in kidney tissue, Immunohistochemical images were retrieved from the Human Protein Atlas (HPA) database. The HPA database is a comprehensive resource for protein expression information, offering a large number of protein expression images for various human tissues and cell types. These images, obtained through immunohistochemistry, allow for the direct visualization of hub gene expression in different tissues and cells [31].

### 2.11. Molecular docking

The 2D structures of 10 air pollutants were retrieved from the PubChem database, and their 3D structures were generated by importing the 2D structures into ChemOffice software. High-resolution crystal structures of hub gene targets were selected from the RCSB PDB database to serve as receptors for molecular docking. The proteins were processed in PyMOL software to remove water and phosphate groups and were saved as PDB files. Molecular docking was carried out using AutoDock Vina 1.5.6 software to investigate the interactions between the proteins and ligands. Interaction visualizations, highlighting the key residues, were created using Discovery Studio 2019 software to generate both 2D and 3D analysis images.

## 3. Results

### 3.1. Identification of air pollution-related targets

**Figure 2.**
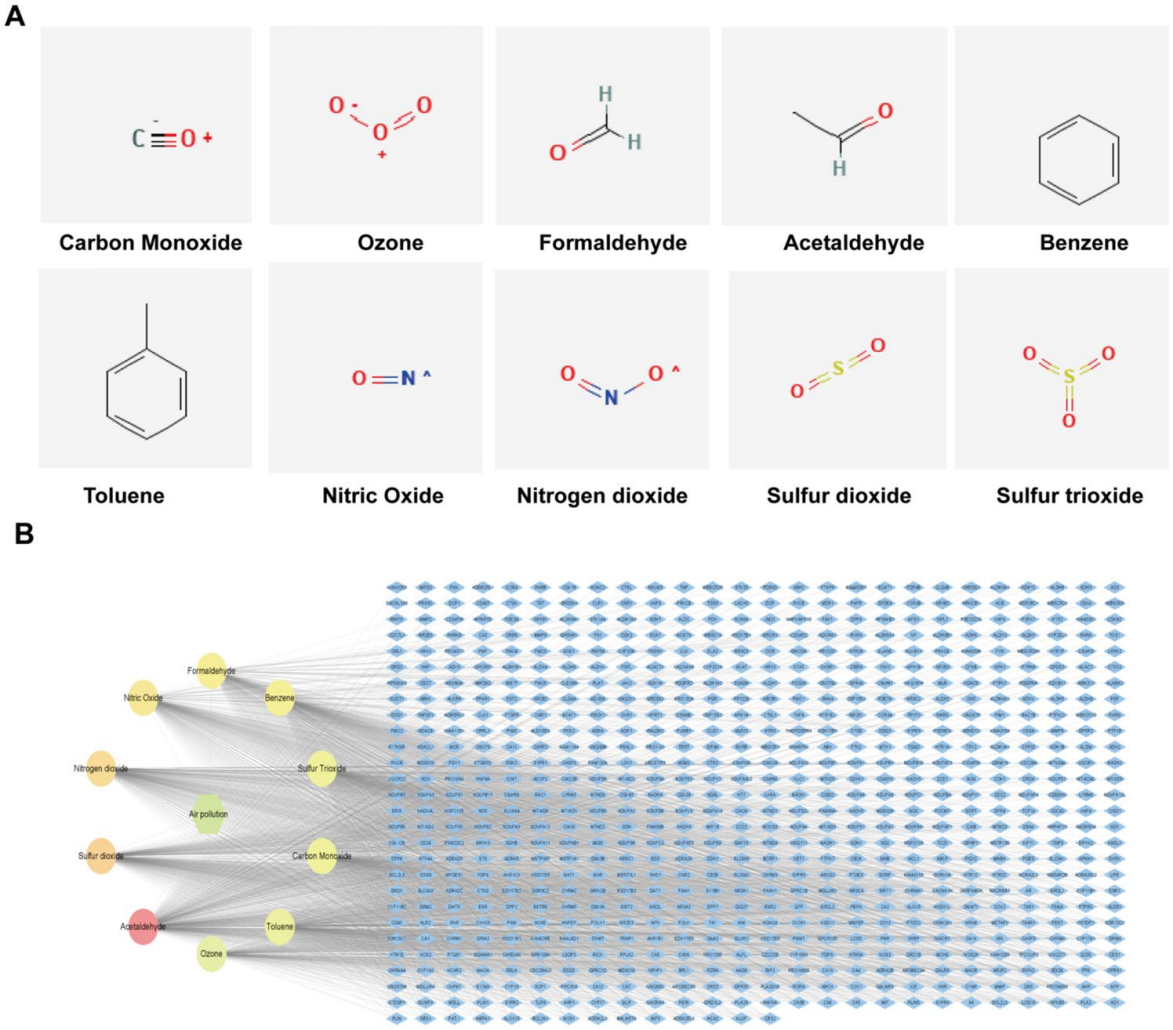
Air pollution-related targets. (A) Structural diagrams of 10 types of air pollutants; (B) Network diagram of air pollutants and their targets (blue diamonds represent targets, circles represent the 10 specific air pollutants, and the central green hexagon represents the air pollutants).

By integrating information from multiple databases, this study identified 714 target proteins from 10 types of air pollutants. These target proteins are defined as ARGs. Table 1 and Figure 2A provide specific information on the air pollutants, with the corresponding target proteins detailed in Table S1. To further explore the interactions between air pollutants and their target proteins, these target proteins were imported into Cytoscape, resulting in the construction of a network diagram containing 727 nodes and 2851 edges (Figure 2B). This network clearly illustrates that air pollutants may exert their toxicological effects through these targets.

**Table 1.** Air pollutant information.

Name	Molecular formula	Molecular weight	SMILES	Target number
Carbon Monoxide	CO	28.010 g/mol	[C−]#[O+]	233
Ozone	O_3_	47.998 g/mol	[O−][O+]=O	201
Formaldehyde	CH_2_O	30.026 g/mol	C = O	267
Acetaldehyde	C_2_H_4_O	44.05g/mol	CC = O	486
Benzene	C_6_H_6_	78.11g/mol	C1 = CC = CC = C1	265
Toluene	C_7_H_8_	92.14g/mol	CC1 = CC = CC = C1	222
Nitric Oxide	NO	30.006 g/mol	[N]=O	284
Nitrogen dioxide	NO_2_	46.006 g/mol	N(=O)[O−]	314
Sulfur dioxide	O_2_S	64.07 g/mol	O = S=O	335
Sulfur trioxide	O_3_S	80.07 g/mol	O = S(=O)=O	234

### 3.2. DKD-related targets

**Figure 3.**
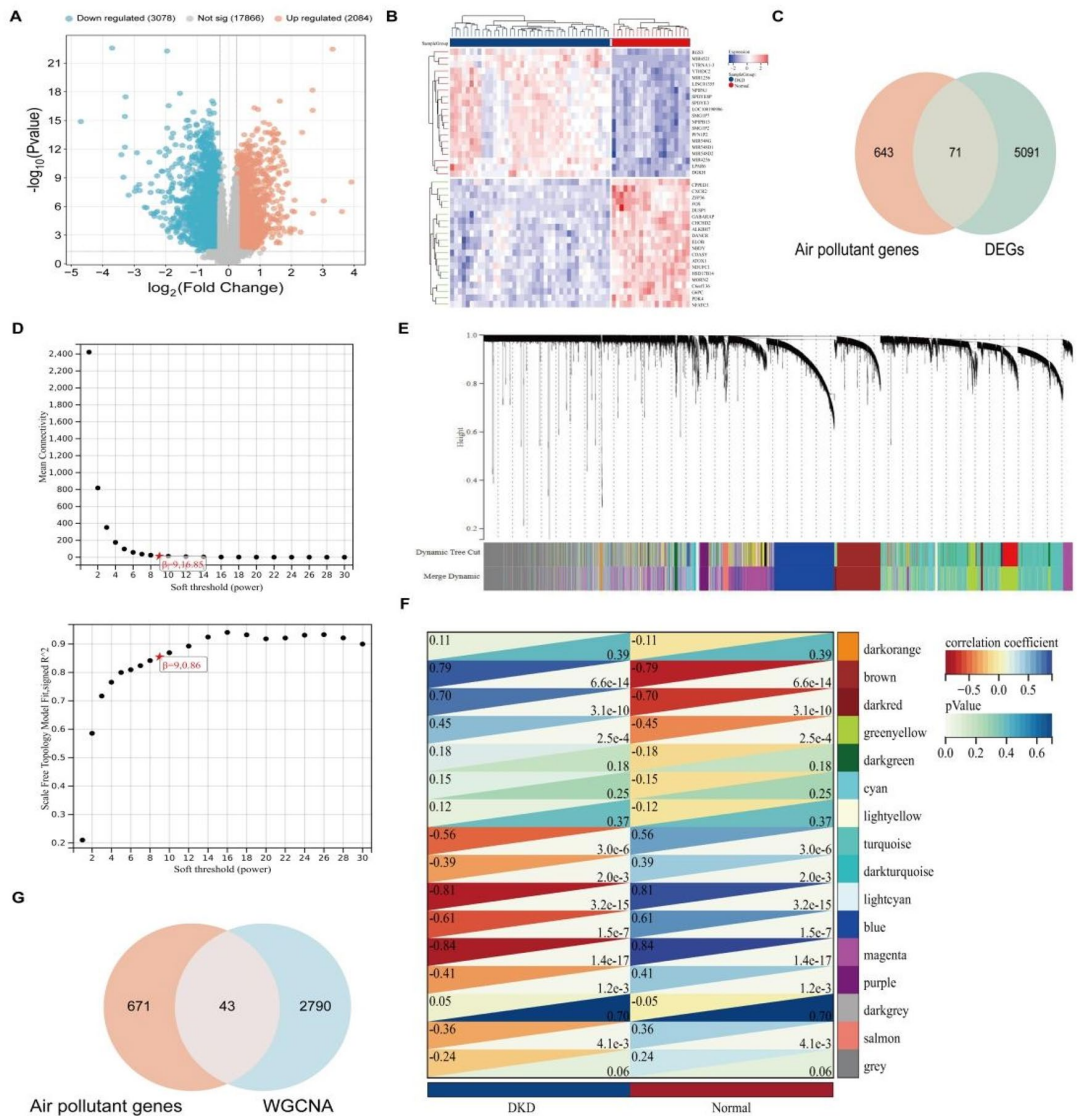
DKD target acquisition. (A) Volcano plot of DEGs; (B) Heatmap of DEGs; (C) Venn of ARGs and DEGs; (D) *β* = 9 was chosen as the soft threshold on the basis of scale independence and average connectivity; (E) Gene coexpression modules in different colors under the gene tree; (F) Heatmap of the correlation between module genes and DKD. The triangle in the upper left indicates the correlation coefficient with color, and the values in the lower right indicate *p* values; (G) Venn diagram of ARGs and module genes.

Using the GSE96804 dataset, potential targets for DKD were identified. Differential expression analysis revealed 3078 downregulated genes and 2084 upregulated genes (Figure 3A,B). The DEGs were then intersected with the ARGs, resulting in the identification of 71 common genes (Figure 3C). In the WGCNA analysis, the optimal soft threshold power was determined to be 9 based on the R^2^ statistic and average connectivity criteria (Figure 3D). Through hierarchical clustering analysis, the gene expression profiles in the GSE96804 dataset were divided into 16 co-expression modules (Figure 3E,F). Among these, the magenta and lightcyan modules showed significant negative correlations with DKD (magenta: *r* = −0.84, *p* = 1.4 × 10–17; lightcyan: *r* = −0.81, *p* = 3.2 × 10–15), while the brown and darkred modules showed positive correlations with DKD (brown: *r* = 0.79, *p* = 6.6 × 10^−14^; darkred: *r* = 0.70, *p* = 3.1 × 10^−10^). After merging the genes from these four modules, 2833 module genes were identified. These were further intersected with the ARGs to obtain 43 common genes (Figure 3G).

### 3.3. Construction of the PPI network and enrichment analysis

**Figure 4.**
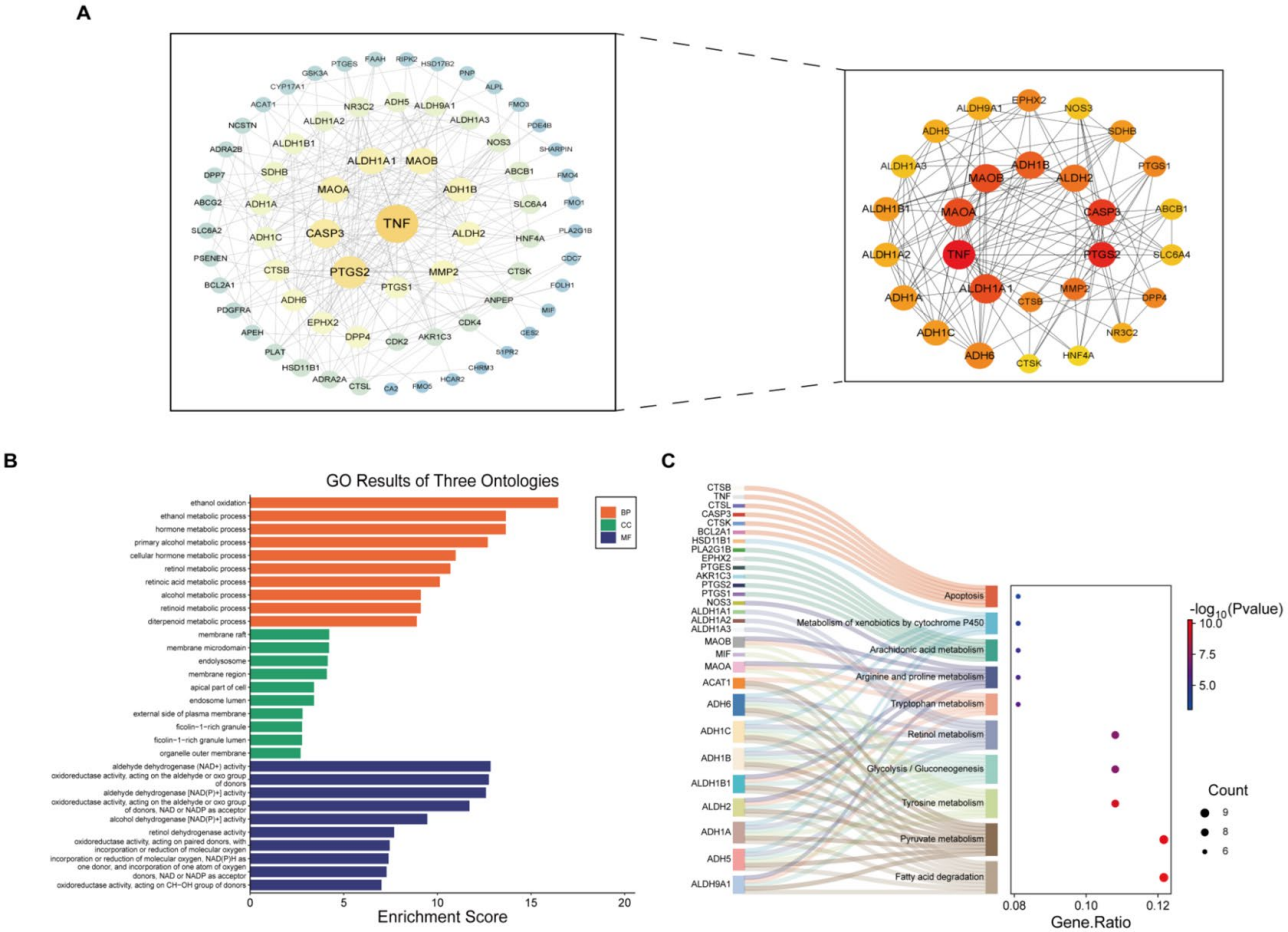
PPI network and enrichment analysis. (A) PPI network (The circles on the left represent 80 genes, while the right side shows the top 28 targets with degree ≥ 8 in the PPI network. The deeper the color and the larger the circle, the higher the degree value); (B) GO enrichment analysis; (C) KEGG enrichment analysis.

To identify the potential targets of air pollutants on DKD, two intersecting gene sets were merged, ultimately identifying 80 genes (Table S2). These genes are considered potential targets through which ten types of air pollutants may influence DKD. The top 28 targets with degree ≥8 in the PPI network were selected for display (Figure 4A). GO and KEGG enrichment analyses were subsequently performed on these targets to explore their biological functions. The GO enrichment analysis revealed that biological processes are primarily involved multiple metabolic pathways, including ethanol oxidation and hormone metabolic processes. Cellular components were mainly enriched in membrane rafts, endosomal lumen, and the outer leaflet of the plasma membrane. Molecular functions were predominantly enriched in aldehyde dehydrogenase (NAD+) activity and oxidoreductase activity acting on donor aldehyde or oxygen groups (Figure 4B). KEGG pathway enrichment primarily involved apoptosis, arachidonic acid metabolism, arginine and proline metabolism, tryptophan metabolism, retinol metabolism, and glycolysis/gluconeogenesis pathways (Figure 4C).

### 3.4. Machine learning-based selection of hub genes

**Figure 5.**
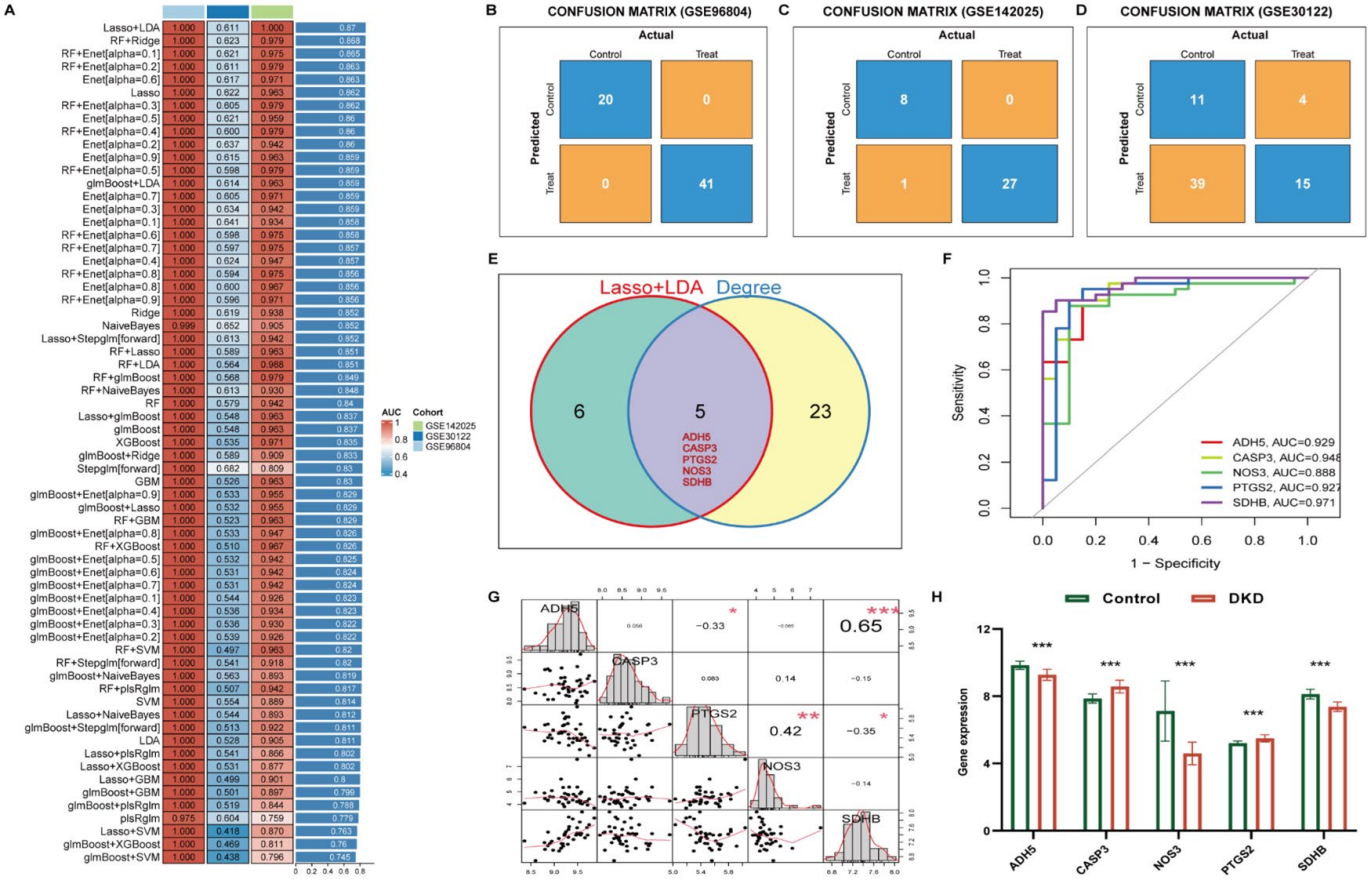
Machine learning-based selection of hub genes. (A) The 113 combinations of prediction models using 10-fold cross-validation with ranked AUC index; (B) Confusion matrix of GSE96804; (C) Confusion matrix of GSE142025; (D) Confusion matrix of GSE30122; (E) Venn diagram of Lasso + LDA and Degree; (F) ROC curve of hub genes (training set); (G) Correlation analysis between hub genes; (H) Box plot of hub genes in training set (*p < 0.05, **p < 0.01, *** *p* < 0.001).

Based on 80 potential target genes, 113 predictive models were constructed using 12 different machine learning methods. In the model performance evaluation, the Lasso + LDA method achieved a peak mean AUC of 0.87 in both the training and testing cohorts, and was consequently identified as the most effective classification model (Figure 5A; Table S3; Figure S1). Further confusion matrix analysis indicated that the model exhibited good predictive performance (Figure 5B–D). The Lasso + LDA model identified 11 candidate genes, and intersection with the 28 genes from the PPI network yielded five hub genes: ADH5, CASP3, NOS3, PTGS2, and SDHB (Figure 5E). To investigate whether these functionally diverse hub genes might be co-regulated in DKD, we performed correlation analysis across the study samples (Figure 5F). Notably, ADH5 and SDHB exhibited the strongest positive correlation (*r* = 0.65, *p* < 0.001), followed by NOS3 and PTGS2 (*r* = 0.42, *p* < 0.01), suggesting potential shared regulatory mechanisms or common upstream influences. The AUC of hub genes ranged from 0.888 to 0.971, suggesting that the hub genes have high diagnostic efficacy in distinguishing DKD from non-DKD (Figure 5G). Specifically, ADH5, NOS3, and SDHB were downregulated in DKD patients, while PTGS2 and CASP3 were upregulated (Figure 5H).

### 3.5. Validation of hub genes and MR analysis

**Figure 6.**
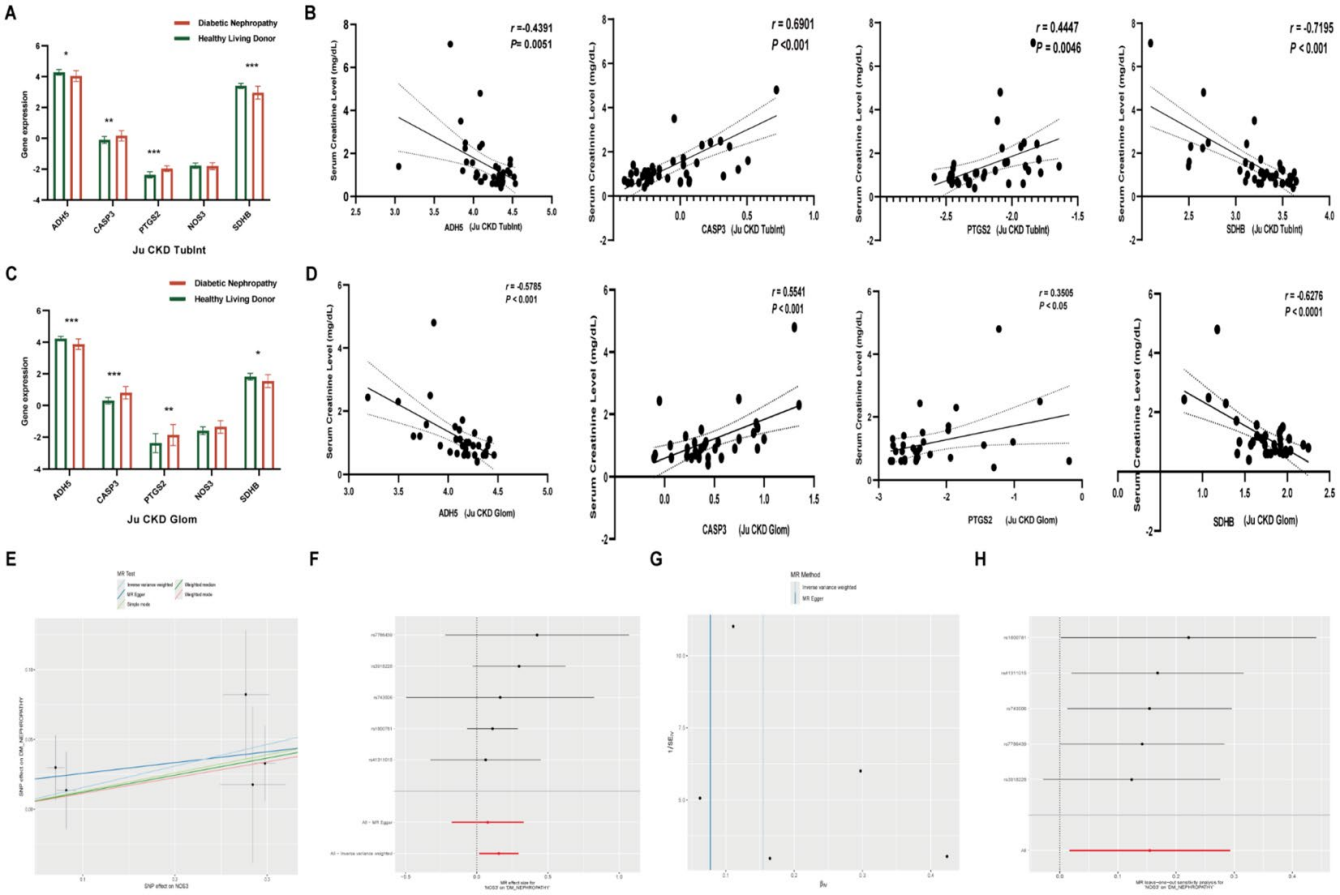
Validation of hub genes and MR analysis. (A) Hub genes in Ju CKD TubInt; (B) Correlation between hub genes and Scr in Ju CKD TubInt; (C) Hub genes in Ju CKD Glom; (D) Correlation between hub genes and Scr in Ju CKD Glom; (E) Scatter plot; (F) Forest plot; (G) Funnel plot; (H) Leave-one-out sensitivity analysis.

To further validate the hub genes, the expression of the 5 hub genes in glomeruli and renal tubules of DKD patients was analyzed using the Nephroseq database. The results showed that both in the glomeruli and renal tubules, ADH5 and SDHB were downregulated, while PTGS2 and CASP3 were upregulated in DKD patients (Figure 6A,C). Furthermore, ADH5 and SDHB were negatively correlated with Scr, while PTGS2 and CASP3 were positively correlated with Scr (Figure 6B,D), further supporting the potential of hub genes as diagnostic biomarkers for DKD. Further MR analysis of hub genes and DKD revealed that among the five hub genes, only NOS3 exhibited a statistically significant causal link to DKD, while the other hub genes did not demonstrate causality. Five independent SNPs (rs7786439, rs3918228, rs743506, rs1800781, and rs41311015) were selected as instrumental variables. The primary analysis method, IVW, revealed that higher expression of NOS3 was strongly linked to an increased risk of DKD (OR = 1.23, 95% CI: 1.05–1.44, *p* = 0.012). The MR-Egger regression intercept was not statistically significant (*p* = 0.621), indicating no evidence of horizontal pleiotropy. The MR-PRESSO global test also did not detect any significant outliers (*p* > 0.05), further supporting the robustness of the results. Additionally, the leave-one-out analysis showed that the overall effect remained consistent when each SNP was removed one at a time, confirming the stability of the findings (Figure 6E–H).

### 3.6. Single-cell analysis

**Figure 7.**
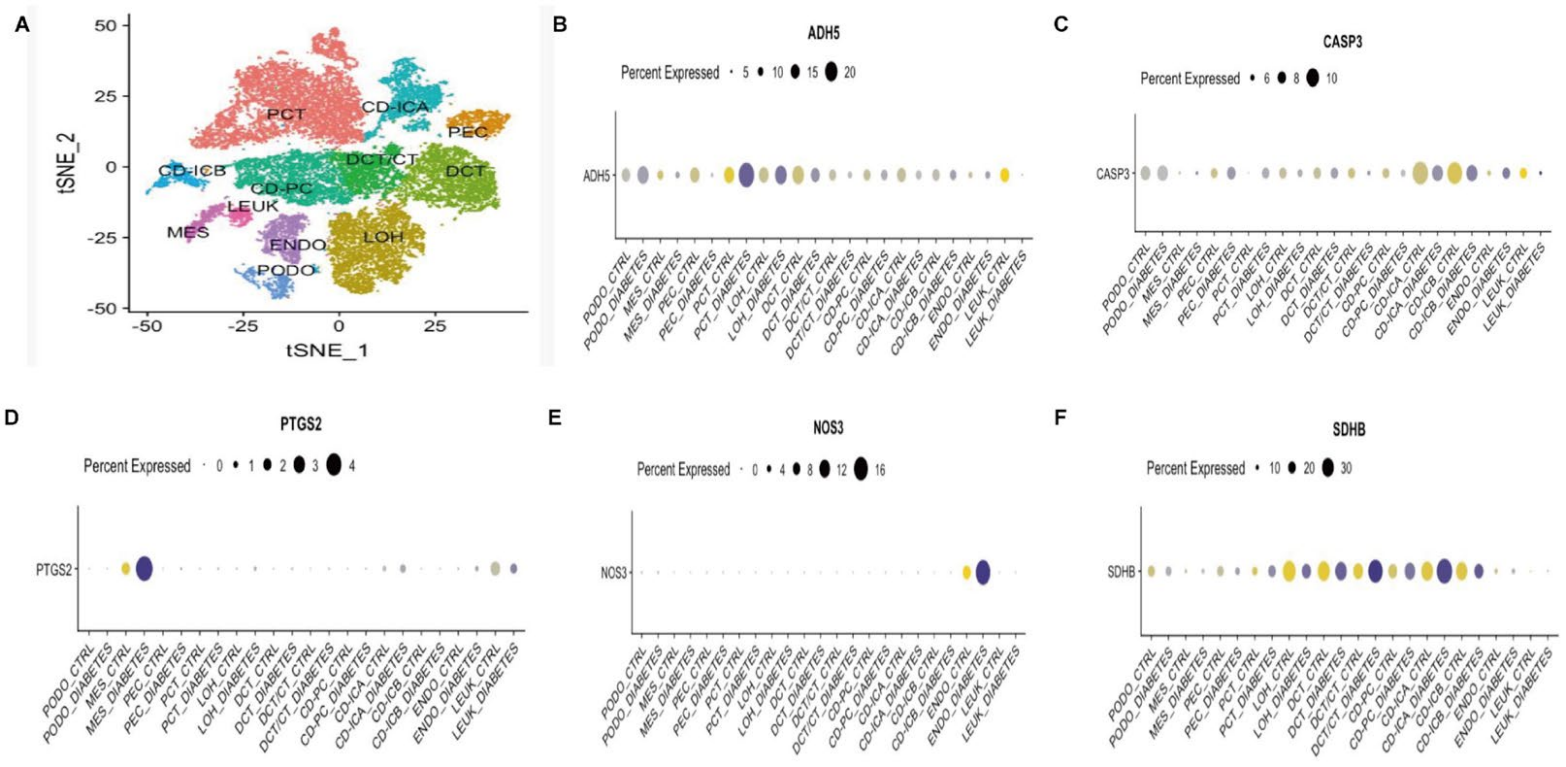
Single-cell analysis. (A) t-SNE plot of single-cell sequencing distribution; (B) Bubble plot of ADH5 expression levels; (C) Bubble plot of CASP3 expression levels; (D) Bubble plot of PTGS2 expression levels; (E) Bubble plot of NOS3 expression levels; (F) Bubble plot of SDHB expression levels.

To further explore the cell-specific expression patterns of hub genes in DKD, single-cell sequencing analysis was performed based on the KIT database. Figure 7A presents the T-SNE plot of single-cell sequencing analysis in DKD, displaying clusters representing 12 distinct cell types. ADH5 is primarily concentrated in PCT and LOH (Figure 7B). CASP3 is primarily concentrated in CD-ICB (Figure 7C). PTGS2 is predominantly distributed in mesangial cells (MES) (Figure 7D). NOS3 is primarily distributed in endothelial cells (ENDO) (Figure 7E), while SDHB is mainly found in distal tubular cells (DCT) and CD-ICA (Figure 7F). The hub genes display unique distribution patterns across different cell types in DKD, which may be closely linked to their involvement in the disease’s pathogenesis.

### 3.7. GSEA analysis

**Figure 8.**
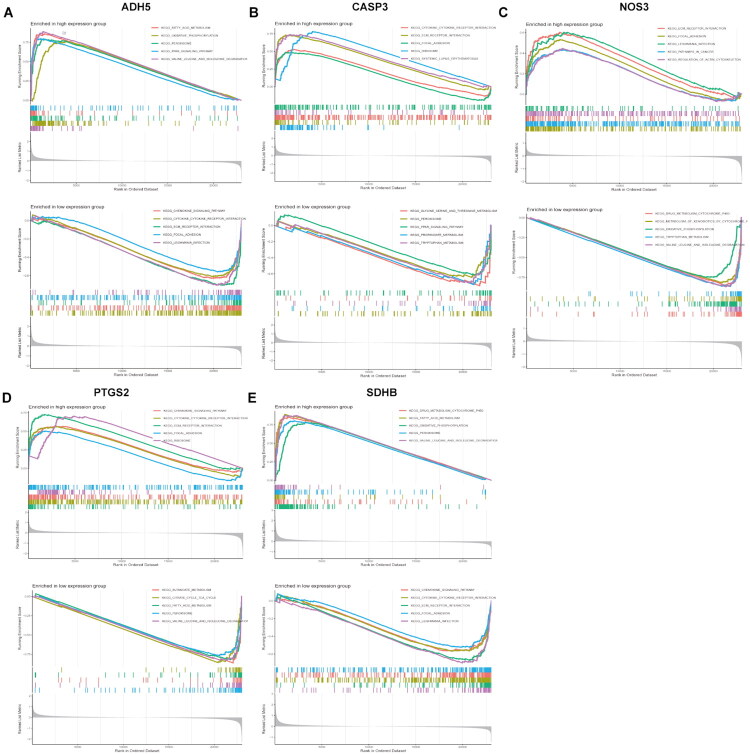
GSEA analysis of hub genes. (A) ADH5; (B) CASP3; (C) NOS3; (D) PTGS2; (E) SDHB. The x-axis represents the rank of genes in the dataset, while the y-axis displays the running enrichment score. Vertical dashed lines indicate the location of the genes within the ranked list.

To further investigate the potential regulatory mechanisms of hub genes in DKD, single-gene GSEA analysis was performed. The analysis results indicated that the expression of hub genes is closely associated with several biological pathways (Figure 8). Specifically, GSEA indicated that samples in the high-ADH5 expression group exhibited significant upregulation of fatty acid metabolism and oxidative phosphorylation pathways, and downregulation of chemokine signaling and extracellular matrix (ECM)-receptor interaction pathways (Figure 8A). In the high-CASP3 expression group, ECM-receptor interaction pathways were significantly upregulated, whereas PPAR signaling and glycine, serine, and threonine metabolism pathways were significantly downregulated (Figure 8B). Samples with high NOS3 expression exhibited upregulation of ECM-receptor interaction and regulation of actin cytoskeleton pathways, and downregulation of oxidative phosphorylation and tryptophan metabolism pathways (Figure 8C). High PTGS2 expression was associated with upregulation of chemokine signaling, and downregulation of butyrate metabolism and citric acid cycle (TCA cycle) pathways (Figure 8D). High SDHB expression was associated with upregulation of cytochrome P450 drug metabolism pathways, and downregulation of chemokine signaling and cytokine–cytokine receptor interaction pathways (Figure 8E). These results suggest that the progression of DKD involves complex biological processes, and the five hub genes may affect DKD development by being associated with pathways related to metabolism, inflammation, and immunity.

### 3.8. Immune infiltration analysis

**Figure 9.**
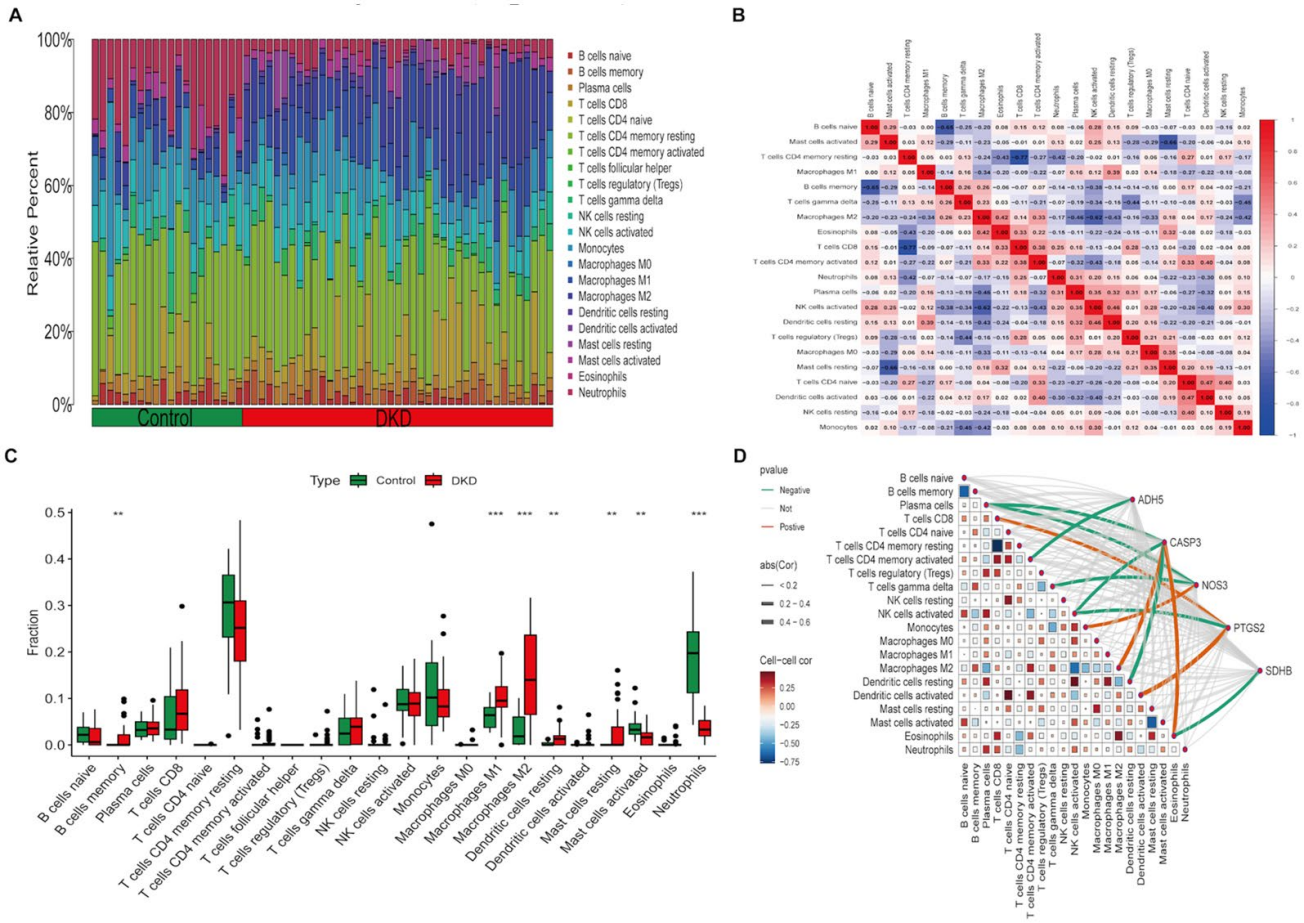
Immune infiltration. (A) Stacked diagram; (B) Correlation heatmap between immune cells; (C) Box plot; (D) Correlation heatmap between hub genes and immune cells. (*p < 0.05, **p < 0.01, *** *p* < 0.001).

The progression of DKD is often accompanied by immune cell infiltration. Immune cells such as macrophages, T lymphocytes, neutrophils, and mast cells play a key role in promoting both the progression and resolution of inflammation through complex signaling pathways and cytokine interactions [32–35]. Previous studies have shown that air pollution can influence changes in the immune microenvironment of the body [36–38]. The GSEA analysis results showed that immune-related signaling pathways were significantly enriched in hub genes. The levels of immune cells were compared based on the CIBERSORT algorithm (Figure 9A). Figure 9B further illustrates the correlation between immune cells. DKD group exhibited significantly higher levels of infiltration, particularly memory B cells, M1 and M2 macrophages, and resting mast cells (Figure 9C). ADH5 is negatively correlated with activated CD4 T cells; CASP3 is negatively correlated with plasma cells and activated NK cells, and positively correlated with M2 macrophages and eosinophils. NOS3 is negatively correlated with plasma cells and γδT cells, and positively correlated with monocytes. PTGS2 is negatively correlated with activated NK cells and positively correlated with activated dendritic cells. SDHB is positively correlated with eosinophils. The results imply that the hub genes could play a role in the initiation and progression of DKD through modulation of immune cell infiltration within the kidney tissue (Figure 9D).

### 3.9. Hub genes expression patterns

**Figure 10.**
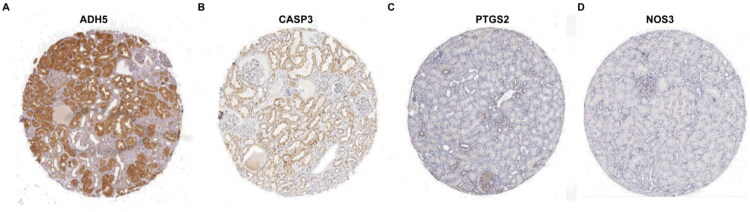
Expression patterns of hub genes. (A) ADH5; (B) CASP3; (C) PTGS2; (D) NOS3.

The proximal tubules and glomeruli serve distinct functions in the pathological development of DKD, and the varied expression of these genes across different regions could be strongly linked to their roles in disease initiation and progression. To further validate the expression patterns of the selected hub genes in renal tissue, we conducted a search in the HPA database. Except for SDHB, which was not found in the HPA database, ADH5 is highly expressed in the proximal tubules and lowly expressed in the glomeruli; CASP3 is mainly expressed in the proximal tubules; NOS3 is lowly expressed in the glomeruli; PTGS2 is highly expressed in the glomeruli and lowly expressed in the proximal tubules (Figure 10).

### 3.10. Molecular docking

**Figure 11.**
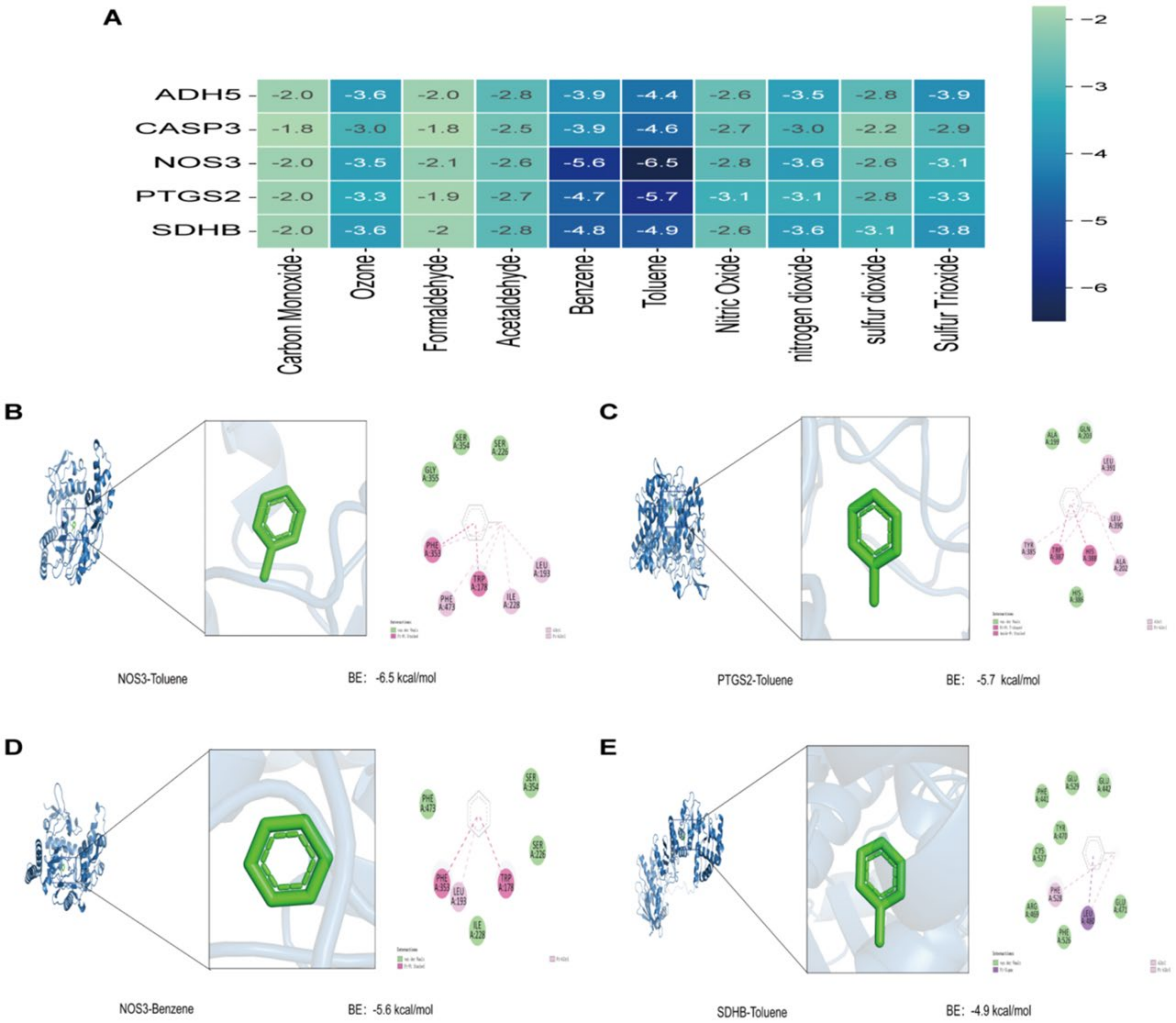
Molecular docking. (A) Heatmap of molecular docking results; (B) Toluene-NOS3; (C) Toluene-PTGS2; (D) Benzene-NOS3; (E) Toluene and SDHB.

To further explore the potential interactions between air pollutants and hub genes, this study performed molecular docking simulations of five hub genes (ADH5, CASP3, NOS3, PTGS2, and SDHB) with ten air pollutants. The binding energy results are shown in Figure 11A. Typically, a binding energy below −5.0 kcal/mol signifies moderate binding affinity, whereas a value lower than −7.0 kcal/mol is indicative of a robust binding interaction. The top four binding energies were selected for display of the binding conformations (Figure 11B–E). The binding energy of NOS3 and Toluene is the highest at −6.5 kcal/mol, with hydrophobic interactions formed between the PHE473, ILE228, and LEU193 residues on the NOS3 receptor and Toluene. The binding energy between Toluene and PTGS2 is −5.7 kcal/mol, with hydrophobic interactions between the TYR385, ALA202, and LEU390 residues on the PTGS2 receptor and Toluene. The binding energy between Benzene and NOS3 is −5.6 kcal/mol, with hydrophobic interactions between the LEU193 residue on the NOS3 receptor and Benzene. The binding energy between SDHB and Toluene is −4.9 kcal/mol, with hydrophobic interactions between the PHE528 residue on the SDHB receptor and Toluene. These molecular docking results indicate that air pollutants, such as Toluene and Benzene, can form stable interactions with the proteins encoded by the hub genes.

## 4. Discussion

The pathogenesis of DKD involves complex mechanisms such as metabolic disorders, hemodynamic abnormalities, inflammation, and fibrosis [39]. Previous studies have primarily focused on the damage to the kidneys caused by hyperglycemia and metabolic toxicity. While this paradigm has made some progress, effective therapeutic strategies are still lacking [40]. The percentage of ESRD patients due to diabetes has increased from 22.1% to 31.3% globally [41], making it critical to explore alternative potential pathogenic mechanisms. Air pollution has become the second leading cause of death worldwide [42], and is an often overlooked risk factor for DKD. In recent years, increasing evidence has shown a significant adverse association between air pollution and the risk of developing T2DM, diabetic complications, and the mortality rate from T2DM or diabetic complications [43].

**Figure 12.**
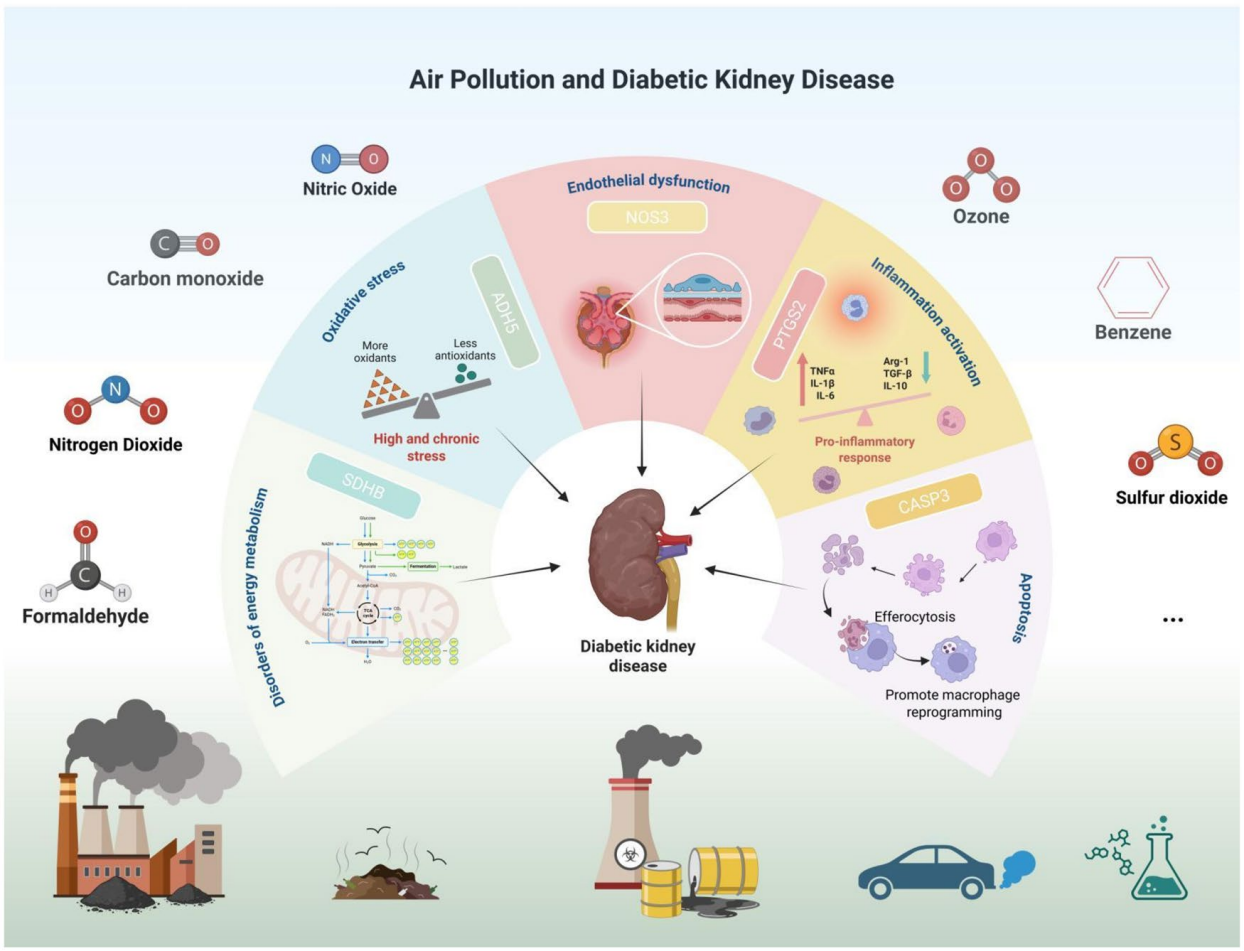
Air pollution and diabetic kidney disease.

Air pollution stands as one of the leading environmental health risks in contemporary society, stemming from numerous sources like industrial emissions (particulate matter and nitrogen oxides emitted by motor vehicles), domestic pollution (coal heating, straw burning), construction and road dust, and agricultural activities (fertilizer volatilization, ammonia emissions from livestock farming) [44]. The kidneys, as an important excretory organ, concentrate environmental toxins during the filtration process, making them particularly susceptible to the toxic effects of environmental pollutants. Previous studies have shown that long-term exposure to gaseous air pollutants such as NO2, CO, O3, and SO2 induces systemic inflammation and oxidative stress, affecting renal vascular endothelial function. This leads to renal vasoconstriction and endothelial dysfunction, which in turn decreases the glomerular filtration rate and increases the risk of chronic kidney disease progression [45]. Moreover, an independent positive correlation has been observed between air pollution and the risk of ESRD in diabetic patients, though the exact mechanism remains unclear [13,15]. This study focuses on the effects of 10 classic air pollutants (such as Carbon Monoxide, Ozone, Formaldehyde, Acetaldehyde, Benzene, etc.) on DKD and elaborates on their impact on DKD and potential molecular targets (Figure 12).

By integrating network toxicology, machine learning, and MR methods, we systematically explored the possible molecular connections between air pollutants and DKD. We ultimately identified five hub genes (ADH5, CASP3, NOS3, PTGS2, and SDHB) that may exhibit significant differences and could suggest diagnostic potential. In the Nephroseq database, except for NOS3, the expression of ADH5, CASP3, PTGS2, and SDHB in both glomeruli and renal tubules appeared consistent with the training set and showed potential correlation with Scr levels. ADH5 is an important detoxifying enzyme primarily involved in the detoxification of formaldehyde and DNA repair processes [46]. It is a key intracellular negative regulator of NLRP3 inflammasome activation [47,48]. Dysfunction of ADH5 leads to obesity-related insulin resistance [49]. We observed downregulation of ADH5 expression in DKD patients, and GSEA analysis revealed that the high-ADH5 expression group exhibited significant upregulation of fatty acid metabolism and oxidative phosphorylation pathways. The progression of DKD is typically marked by the exacerbation of renal fibrosis caused by impaired fatty acid oxidation within the renal tubules [50]. Activation of PPARα signaling can alleviate lipid accumulation and inflammation in DKD [51]. The downregulation of ADH5 expression increases the risk of destructive effects from formaldehyde exposure in DKD patients, where the accumulation of toxic aldehydes and disruptions in energy metabolism may exacerbate kidney damage [52,53]. Single-cell analysis indicated that the high expression of ADH5 in proximal tubule cells may be associated with renal tubular damage. Immunohistochemical analysis further revealed that ADH5 is predominantly highly expressed in the renal tubules, which is consistent with its functional characteristics.

CASP3 is a key executioner protease in apoptosis [54] and is upregulated in DKD patients [55]. Immune infiltration analysis revealed a positive correlation between CASP3 expression and M2 macrophage infiltration. CASP3-dependent apoptosis is associated with renal tubular cell death under diabetic conditions [56,57]. Apoptotic cells or cell debris have strong chemotactic effects on macrophages [58], and the loss of CASP3 alleviates renal tubular damage and inflammation, ultimately preventing the development of renal fibrosis [59]. This suggests that air pollutants may promote renal tubular epithelial cell apoptosis by activating CASP3, while also inducing M2 macrophage infiltration, thus contributing to the progression of renal fibrosis. Increased accumulation of ECM is a key feature of DKD, contributing to thickening of the glomerular and tubular basement membranes, mesangial expansion, and tubulointerstitial fibrosis [60]. Inhibition of ECM receptor interactions in glomerular cells can alleviate DKD [61]. GSEA analysis revealed that CASP3 is associated with the upregulation of ECM-receptor interaction pathways, further supporting this hypothesis. Furthermore, important risk factors for DKD, such as blood glucose fluctuations [62], insulin resistance [63], and advanced glycation end products [64], promote the activation of CASP3 and accelerate the progression of DKD.

Endothelial dysfunction is closely associated with diabetic microvascular complications, including DKD. Impairment of NOS3 function or activity, along with abnormal downstream NO signaling, is closely associated with the onset of endothelial dysfunction [65]. NOS3 catalyzes the production of NO, a key molecule that functions as a vasodilator and anti-inflammatory mediator, playing a crucial role in regulating microvascular tone and immune responses [66]. Previous studies have confirmed that atmospheric pollutant SO_2_ can interfere with the endothelial PI3K/Akt/eNOS and NO/cGMP signaling pathways, thereby affecting the normal regulation of vasodilation [67]. NOS3 plays a key role in regulating vascular endothelial function, and dysfunction of NOS3 in glomerular endothelial cells has been shown to play a crucial role in the progression of DKD [68,69]. NOS3-deficient mice exhibit kidney pathology consistent with diffuse glomerulosclerosis observed in late-stage DKD [70]. Insulin resistance enhances vascular eNOS activity [71], while overexpression of NOS3 in the kidneys can suppress insulin resistance in obesity-related glomerulopathy [72]. Single-cell analysis further revealed high expression of NOS3 in endothelial cells, suggesting that NOS3 may be related to endothelial dysfunction. GSEA analysis indicated that NOS3 is associated with ECM-receptor interaction and actin cytoskeleton regulation. Rearrangement of the actin cytoskeleton in podocytes is considered a key process in the development of glomerular injury [73]. The formation of the F-actin cytoskeleton and the assembly of focal adhesions may disrupt podocyte barrier function, resulting in proteinuria [74].

Mendelian randomization results confirmed a causal relationship between NOS3 and DKD. Further molecular docking results showed that toluene and benzene can form stable interactions with NOS3, providing molecular evidence for the direct impact of air pollutants on NOS3 function.

PTGS2, also known as COX-2, is a key regulator of the inflammatory response. It catalyzes the conversion of arachidonic acid to prostaglandin endoperoxides, thereby regulating pain, fever, and inflammation [75]. Inflammatory immune response is a crucial mechanism in the pathogenesis of DKD. Chemokines and their receptors play pivotal roles in the recruitment and differentiation of inflammatory immune cells. Chemokines are thus potential therapeutic targets and may also serve as biomarkers for DKD [76,77]. In DKD animal models, COX-2 expression in kidney tissues is increased. Inhibition of COX-2 expression alleviates renal inflammation, oxidative stress, and fibrosis, delaying the progression of DKD [78–81]. The HPA database shows that PTGS2 is predominantly highly expressed in the glomeruli. Molecular docking results indicate that toluene can form hydrophobic interactions with PTGS2, increasing the risk of interference with glomerular inflammation or oxidative stress pathways.

SDHB is a subunit of mitochondrial respiratory chain complex II (succinate dehydrogenase, SDH), playing a crucial role in maintaining the normal operation of the mitochondrial electron transport chain and the tricarboxylic acid cycle [82]. We found that SDHB expression is downregulated in DKD patients. Air pollutants may affect SDHB expression or activity, disrupting mitochondrial energy metabolism and ultimately promoting kidney damage. Molecular docking results indicate that air pollutants can form stable interactions with the proteins encoded by hub genes.

Molecular docking simulations have identified several interaction residues located within the key functional regions of the proteins. For example, in NOS3, PHE473 and LEU193 are situated within the oxidase domain, near the heme and substrate L-arginine binding pocket, while ILE228 is close to the tetrahydrobiopterin (BH4) cofactor binding site, all of which are crucial for nitric oxide synthesis. In PTGS2, TYR385 is a recognized important catalytic residue in the cyclooxygenase reaction, directly involved in the peroxidation of arachidonic acid. Therefore, the binding of air pollutants to these critical functional regions may directly or indirectly influence the enzyme activity or structural stability of the associated proteins, thereby altering the oxidative balance and inflammatory response within cells.

This study reveals, through multi-omics integration analysis, that air pollutants may influence various biological processes, including metabolic reprogramming, inflammation activation, and immune microenvironment alteration, by acting on key genes such as ADH5, CASP3, NOS3, PTGS2, and SDHB, thereby promoting the onset and progression of DKD. However, there are several limitations: Firstly, this study is based on publicly available transcriptomic and bioinformatics analyses. Due to the lack of experimental validation, the results presented here cannot establish causality and require further verification through independent samples and *in vivo*/*in vitro* experiments. Secondly, we only considered ten common air pollutants, while the actual environment may contain more complex mixtures of pollutants. Thirdly, transcript levels do not fully represent protein expression or enzymatic activity, and sample heterogeneity and batch effects may influence the robustness of the results. Future studies could validate the regulatory mechanisms of pollutants on key genes such as NOS3 through *in vitro* experiments (e.g. exposing HK-2 cells to PM2.5). Additionally, qPCR validation of key gene expression in kidney samples from patients with different pollution exposure levels could be conducted to explore the feasibility of targeted intervention strategies.

## 5. Conclusion

This study systematically suggests ADH5, CASP3, NOS3, PTGS2, and SDHB as key genes through network toxicology, machine learning, and Mendelian randomization, and suggests the potential molecular mechanisms involving metabolic disorders, inflammation, and immune microenvironment alterations through which they contribute to DKD. Air pollutants may act as significant effect amplifiers or independent risk factors, triggering systemic inflammation and oxidative stress, thereby magnifying the damage caused by hyperglycemia and AGEs. These results may enhance our comprehension of the molecular mechanisms linking air pollution to DKD and provide insights that could potentially assist in identifying intervention targets in DKD.

## Supplementary Material

Supplemental Material

## Data Availability

The data that support the findings of this study are openly available in Zenodo at https://doi.org/10.5281/zenodo.19665782, reference number 19665782.
