# Supplementary material for: Unveiling the role of air pollution in diabetic kidney disease: an integrated study combining network toxicology, machine learning, and Mendelian randomization
Source: Ren Fail. 2026 May 24;48(1):2668270. doi: 10.1080/0886022X.2026.2668270 (PMC13202638; doi:10.1080/0886022X.2026.2668270)
Supplement: Supplemental Material [file IRNF_A_2668270_SM0501.docx]

**Supplementary material**


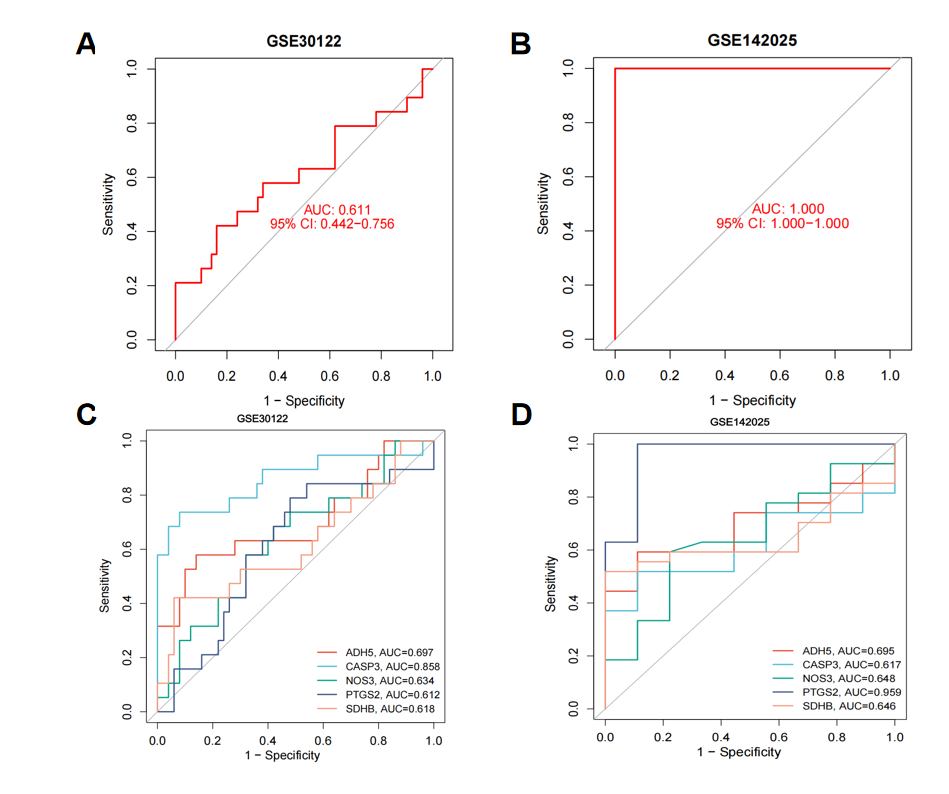


**Figure S1 ROC curves for Lasso+LDA models on test data.** (A)GSE30122; (B) GSE142025; (C-D) ROC curves of hub genes. (C)GSE30122; (D) GSE142025.
